# Improving Care for Spanish-Speaking Older Adults with Breast Cancer: Feasibility, Reliability, and Validity of a Self-Administered Spanish Language Geriatric Assessment

**DOI:** 10.3390/cancers13112685

**Published:** 2021-05-29

**Authors:** Enrique Soto-Perez-de-Celis, Jessica Vazquez, Heeyoung Kim, Can-Lan Sun, Kemeberly Charles, Ashley Celis, Vani Katheria, Daneng Li, William Dale, Mina S. Sedrak

**Affiliations:** 1 Salvador Zubirán National Institute of Medical Science and Nutrition, Vasco de Quiroga 15, Colonia Belisario Domínguez Sección XVI, Tlalpan, Mexico City 14080, Mexico; enrique.sotop@incmnsz.mx; 2 City of Hope Comprehensive Cancer Center, 1500 E Duarte Rd., Duarte, CA 91010, USA; jv20384@usc.edu (J.V.); hekim@coh.org (H.K.); casun@coh.org (C.-L.S.); kecharle@health.ucsd.edu (K.C.); acelis@coh.org (A.C.); vkatheria@coh.org (V.K.); danli@coh.org (D.L.); wdale@coh.org (W.D.)

**Keywords:** geriatric assessment, neoplasms, breast neoplasms, older adults, feasibility studies, validation study, hispanic americans, educational statu

## Abstract

**Simple Summary:**

Conducting a geriatric assessment represents the standard of care for the management of older adults with cancer. However, most studies of the geriatric assessment in oncology have included non-Hispanic white populations with high educational levels living in developed countries. In this study, we assessed the feasibility, reliability, and validity of two methods of administration (electronic touchscreen tablet and paper/pencil) of the Spanish language version of a self-administered geriatric assessment among older women with breast cancer in the United States. Our results show that implementing a self-administered geriatric assessment using either an electronic tablet or paper/pencil is feasible, reliable, and valid in Spanish-speaking older adults. However, in order to complete the geriatric assessment, participants with lower educational levels were more likely to need help and took significantly longer to do so. This study highlights the importance of tailoring assessments and questionnaires to the cultural, social, and educational level of older adults with cancer.

**Abstract:**

We evaluated the feasibility, reliability, and validity of a Spanish-language self-administered geriatric assessment (GA) in older (age ≥ 65) Spanish-speaking women with breast cancer in the United States. Eligible participants (*n* = 181) were recruited and randomized. Feasibility was defined as the participant’s unassisted GA completion rate, completion time, and perception on ease of completion. Reliability and validity were assessed using Spearman’s correlation coefficients. Two-sided *p* < 0.05 was considered significant. Ninety-eight percent of participants (*n* = 177) completed the GA at least once. Median age was 70 years (range: 65–95) and 55% had ≤8th grade education. Forty-one percent (*n* = 73) were unable to complete the GA unassisted, median completion time was 28 min (range 8–90), and 77% (*n* = 136) rated the GA as “easy”/“very easy”. Patients with ≤8th grade education took longer to complete the GA (30 vs. 25 min, *p* = 0.0036) and needed more assistance (59% vs. 19%, *p* < 0.001) than those with ≥9th grade education. Test–retest reliability was high (≥0.82) for all domains except social activity (0.73). Validity among similar scales was found. The self-administered GA is a feasible, reliable, and valid tool for Spanish-speaking older women with breast cancer. Tailoring GA tools to the patients’ educational level is important when implementing tools in multicultural environments.

## 1. Introduction

Considerable global efforts have demonstrated the importance of incorporating the geriatric assessment (GA) as part of the routine care of older adults with cancer [1]. The GA is a multidimensional, comprehensive, and validated clinical tool that evaluates important domains for older adults, including physical function, comorbidity, cognition, nutritional status, polypharmacy, social support, and psychological status [2,3]. Evaluating older adults with cancer using the GA is useful in predicting treatment toxicity, estimating survival, and identifying areas of vulnerability that can be targeted with interventions [4,5,6].

Most studies assessing the feasibility and reliability of the GA in oncology have included older adults with cancer living in high-income countries, who are more likely to have higher health literacy and educational levels, and data regarding its use in populations from developing countries and/or with lower educational levels is lacking. While this is certainly relevant for the implementation of provider-administered GA tools, it is arguably even more so for self-administered assessments, which depend on the patient’s ability to understand and answer questions correctly [7]. One of the first self-administered GA tools for oncology was developed by the Cancer and Aging Research Group (CARG) [8,9], who demonstrated that its implementation in both paper/pencil and electronic touchscreen formats was feasible, reliable, and valid among English-speaking older adults in the US [10]. While this self-administered GA has been translated into multiple languages, including Spanish, the feasibility and reliability of these translated versions is unknown.

Studying the validity of self-administered tools across languages and cultures is highly relevant. Among these, the cross-cultural validation of Spanish language tools is particularly important both for the evaluation of immigrants to high-income nations (4 million older adults in the US self-identify as Hispanic/Latino [11]) and for their implementation in Spanish-speaking countries worldwide. Therefore, the primary objective of this study was assessing the feasibility, reliability, and validity of the Spanish version of the CARG self-administered GA in Spanish-speaking older women with breast cancer living in the US, and the secondary objective was identifying the preferred method of delivering the GA (paper/pencil or electronic touchscreen).

## 2. Materials and Methods

### 2.1. Study Population

Participants were recruited from the outpatient breast cancer clinic at City of Hope, an NCI-designated comprehensive cancer center in Duarte, California between 22 February 2017 and 30 August 2019. Eligible participants were aged ≥ 65 years, had a history of breast cancer (any stage, treatment, and time from diagnosis), and were fluent in Spanish (first or second language). Participants with any performance status could enroll; however, those with significant visual/hearing impairments precluding the ability to read the questions or hear instructions were ineligible. The study was approved by the City of Hope Institutional Review Board (reference number 16,335), and all participants provided written informed consent.

**Figure 1:**
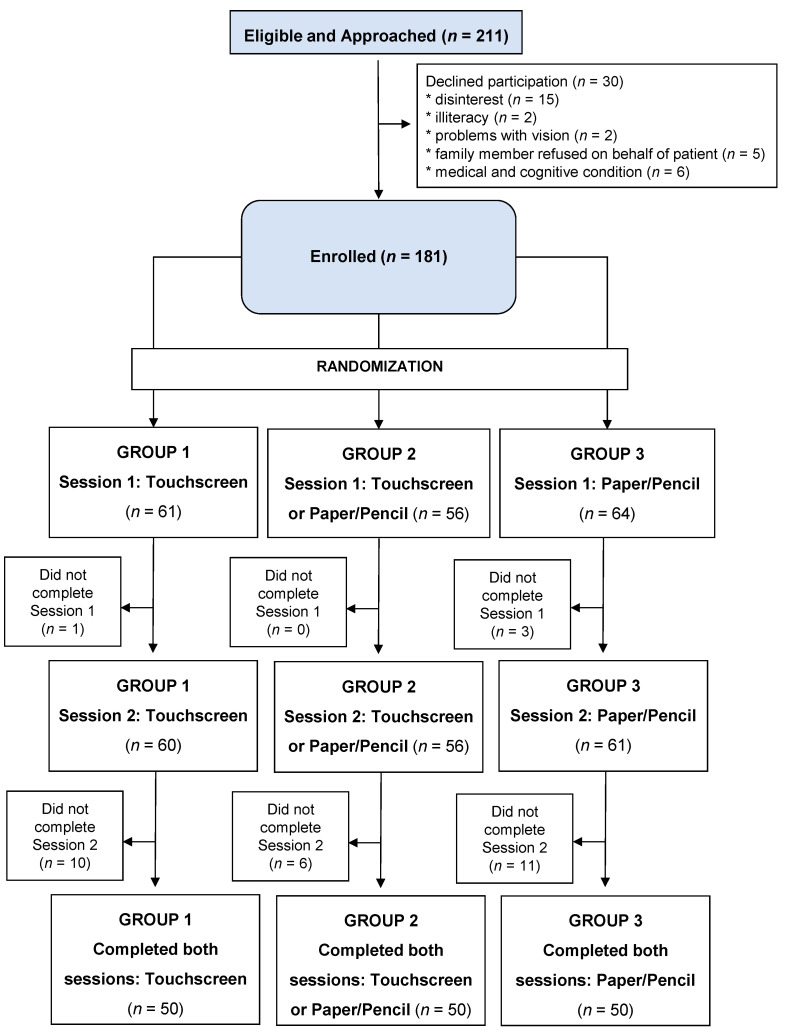
CONSORT diagram.

A total of 211 eligible patients were approached by the research team (Figure 1). Of these, 86% (*n* = 181) agreed to participate. Reasons for refusal included disinterest (*n* = 15), concerns regarding ability to understand questions (*n* = 2), visual fatigue (*n* = 2), family members refusing on behalf of the patient (*n* = 5), and medical or cognitive condition (*n* = 6). Out of the 181 participants who agreed to participate, 98% (*n* = 177) completed session 1 and were evaluable for feasibility outcomes, while 82% (*n* = 150) completed both sessions 1 and 2 and were evaluable for reliability and validity outcomes.

### 2.2. Spanish Version of the Geriatric Assessment (GA)

The English version of the GA was translated into Spanish, and its contents validated using the Functional Assessment of Chronic Illness Therapy (FACIT) Translation and Linguistic Validation Service. A full description of the utilized English-version GA, as well as the reliability and validity of each of its components, has been previously reported [2,3,8,9].

The GA consists of a self-administered portion completed by the patient, followed by a brief portion completed by a research team member evaluating cognition and physical performance. This study focused on the feasibility, reliability, and validity of the patient portion of the GA. The following measures were included in the GA used in this study: (1) Instrumental Activities of Daily Living (IADL) (subscale of the Older Americans Resources and Services [OARS] survey); (2) Activities of Daily Living (ADL) (subscale of Medical Outcomes Survey [MOS] Physical Health Measure); (3) Karnofsky Performance Status (KPS); (4) Number of falls in the past six months; (5) MOS Social Activity Limitations Measure; (6) number of comorbidities (OARS); (7) Mental Health Inventory (MHI-17); (8) MOS Social Support: emotional and tangible subscales; (9) Body Mass Index (BMI); and (10) cognition screening using the Blessed Orientation-Memory Concentration screening test (Table 1).

### 2.3. Study Procedure and Measures

To assess feasibility and reliability, participants were asked to complete the Spanish version of the GA twice within the same day (sessions 1 and 2). To examine various methods of delivering the GA (electronic touchscreen tablet or paper/pencil), participants were randomized into one of three groups: group 1, completion of the touchscreen version in both sessions; group 2, completion of the touchscreen version and the paper/pencil version (random order); and group 3, completion of the paper/pencil version in both sessions.

In all groups, before starting session 1, participants were given a brief introduction about the GA. Patients randomized to the touchscreen method also received guidance on its use. Participants completed the GA in a quiet room within the clinic space. A Spanish-speaking research assistant or a certified Spanish translator was available if needed. Family members could be in the room with the participant and assist when needed.

At the end of each session, participants were asked to provide feedback about questions that were difficult to understand or perceived to be missing from the survey using open-ended questions; Likert-style items were utilized to assess their perception of the survey length (“too long”, “too short”, or “just right”) and whether any of the questions were upsetting. Participants were asked to rate the difficulty level (“very easy”, “easy”, “difficult”, or “very difficult”) of the GA, their computer skill level (none, beginner, intermediate, or advanced), and to specify their preferred method of GA completion (touchscreen vs. paper/pencil).

Sociodemographic information was self-reported through a study-specific questionnaire which included age, primary language, marital status, educational level, employment status, income, and country of birth. The patient’s breast cancer stage and prior treatment (surgery, radiation, chemotherapy, hormone therapy, and/or systemic therapy) was obtained from chart reviews.

### 2.4. Statistical Analysis

The feasibility of the Spanish version of the GA was evaluated among all patients who completed session 1 (*n* = 177). Feasibility was demonstrated through the following factors: (a) rate of completion of the GA, (b) rate of completion of the GA without assistance from research team members and/or family members, (c) number of questions missed/skipped or selected as “prefer not to answer” or “I don’t know”, (d) length of time needed to complete the GA, (e) patients’ perception of the ease of completing the GA (rating it as “easy”/”very easy” to complete), and (f) patients’ perception of how long it took to complete the GA.

Test–retest reliability for the same method and across methodologies (touchscreen vs. paper/pencil) was evaluated among the 150 participants who completed both sessions using Spearman correlation coefficients. The internal consistency of each component of the GA was analyzed using Cronbach’s alpha coefficient across all surveys, including the three groups and both timepoints (*n* = 300). Spearman correlation coefficients among similar scales were assessed to determine scale validity.

For all analyses, summary statistics including frequencies and percentages were presented for categorical variables and medians with ranges for continuous variables. Chi-square tests/Fisher’s exact method (for categorical variables) and Kruskal–Wallis tests (for continuous variables) were used for between-group comparisons. Feasibility and reliability were assessed for all participants and separately by method (touchscreen vs. paper/pencil). All analyses were performed using SAS 9.4 and a two-sided *p*-value <0.05 considered as statistically significant.

## 3. Results

### 3.1. Patient Demographics and Disease Characteristics

Participants had a median age of 70 years (range 65–95 years). All of them were women; 89% listed Spanish as their primary language and 78% exclusively spoke Spanish at home (Table 2). Most participants were born outside the US, with 67% being from Mexico, 18% from Central America and the Caribbean, and 7% from South America. More than half of the participants (55%) had ≤8th grade education and 33% had graduated high school and/or completed some post-high school education. Most participants were married (45%), retired (52%), and had an annual household income of <$50,000 (83%). Most had early-stage breast cancer and had received prior treatment, including surgery, radiation, chemotherapy, and/or hormonal therapy. There were no significant differences between groups (all *p* > 0.05).

### 3.2. Feasibility 

Among the 177 participants who completed session 1, 87 used the touchscreen version of the GA and 90 the paper/pencil version (Table 3). Fifty-nine percent (*n* = 104) were able to complete the GA on their own, without help from family members or research team members. Completion rates without requiring assistance were similar between touchscreen and paper/pencil methods (56% vs. 61%, *p* = 0.52). Common reasons for needing assistance included difficulty understanding questions (*n* = 27), visual problems (*n* = 23), fatigue (*n* = 7), being uncomfortable with the technology/touchscreen format (*n* = 7), and literacy (*n* = 3). Participants with ≤8th grade educational level were more likely to need assistance than those with ≥9th grade educational level (59% vs. 19%, *p* < 0.001). Only one participant left a question unanswered using the touchscreen method, while 27 of those using the paper/pencil method left ≥1 unanswered question (range 1–11, 15 missed one question, six missed two, one missed three, two missed four, one missed five, and two missed 11).

#### 3.2.1. Completion Time

**Figure 2:**
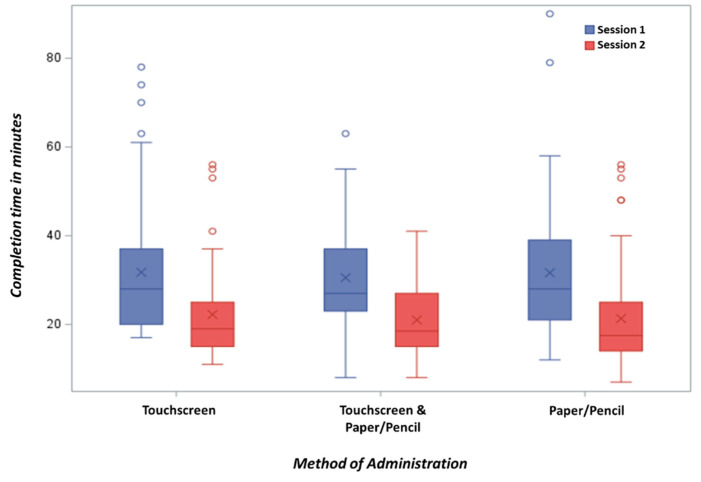
Distribution of completion time for the Spanish-Language Geriatric Assessment by Group. X represents the mean and the horizontal line inside the box represents the median.

Median time to complete the GA was 28 min (range 8–90 min). No differences were observed between the two methods (Table 3; Figure 2). Participants with ≤8th grade educational level took significantly longer to complete the GA than those with a ≥9th grade educational level (median 30 vs. 25 min, *p* = 0.004).

#### 3.2.2. Satisfaction with the GA

Most (76%) of the participants thought the length of the GA was “just right” and the majority (77%) rated the GA as “easy”/“very easy” to complete. Again, no significant differences were observed between the two methods (Table 3). Most participants rated their computer skill level as none/beginner (85%), with none of them rating it as “advanced”. Among those who were randomized to the touchscreen method, more than half (58%) preferred touchscreen technology. However, among those who were randomized to the paper/pencil method, only 36% preferred using touchscreen technology (*p* = 0.005).

### 3.3. Reliability and Validity

One-hundred-and-fifty patients completed both sessions, 50 in each of the three groups (Figure 1). The reliability of the following measures was evaluated and presented in Table 4: IADL; ADL; KPS; MHI-17; MOS Social Activity Limitations Measure; and MOS Social Support. Internal consistency as measured by the Cronbach Alpha in all the surveys completed by the three groups at both timepoints (*n* = 300) was good for all measures (≥0.88), except for MOS Social Activity Limitations Measure (0.68). The test–retest reliability (measured using Spearman’s rank correlation coefficient) of all six scales within the same method (touchscreen and paper/pencil) and across methodologies (touchscreen vs. paper/pencil) was strong (>0.8) except for MOS Social Activity Limitations Measure (>0.7).

The validity of the GA was evaluated for the overall cohort and for each method (Table 5). Correlations between IADL and ADL scores were strong overall (0.75) and >0.72 for all groups. The patient’s assessment of Karnofsky Performance Status (KPS) correlated weakly with the IADL score (0.63) and with the patient’s social activity level (0.47). The MOS Social Activity Limitations score was weakly correlated with ADL (0.55) and IADL (0.47) score.

## 4. Discussion

The Spanish language version of the CARG self-administered GA is feasible, reliable, and valid for obtaining data regarding the relevant geriatric domains among Spanish-speaking older women with breast cancer in the US, both in its touchscreen and paper/pencil version. Most participants were able to complete the GA, and over half were able to do so unassisted, with a median completion time of <30 min. Furthermore, three-quarters rated the GA as “easy” or “very easy” to complete, and most were satisfied with its length. No significant differences in completion times were found between the touchscreen and paper/pencil methods, and both were reliably consistent. Importantly, participants with ≥9th grade education needed less assistance and completed the GA tool faster. The touchscreen method was better at avoiding missed questions, and participants who were exposed to it expressed their preference towards touchscreen technology.

Developing GA tools in Spanish is essential to assess older Spanish-speaking immigrants to high-income nations such as the US, as well as for improving access to geriatric oncology care in low- and middle-income nations in Latin America and the Caribbean. Spanish is the fourth most widely spoken language in the world, with over 500 million speakers, and is the official language of 20 countries in four continents [12]. In addition, it is the most spoken unofficial language in the US, with 13.5% of the US population speaking Spanish at home [13]. Increasing the availability of tools that can be utilized to assess patients in their own language is of the utmost importance for reducing inequities in access to care, including a lack of access to a GA as part of the cancer care of older adults.

However, there are several caveats that must be considered when trying to translate and implement self-administered surveys. This can be shown by analyzing the contrasts between our current findings and our previously published study examining the feasibility, reliability, and validity of the English-language version of the CARG self-administered GA. In that study, where nearly 80% of respondents were non-Hispanic white, the median time to complete the GA was ≤21 min depending on methodology, and <10% of participants needed assistance [10]. We believe that the differences in findings with the present study may be explained by social, economic, and educational factors, rather than by ethnicity or language. In the present study, only 34% of participants had high school or higher education, compared with 96% of participants in the English-language feasibility study. This closely reflects the real-world circumstances of older Hispanic adults in the US, who often have lower educational levels than their non-Hispanic white counterparts, which may be a direct consequence of the low educational coverage in their countries of origin [14,15]. For example, among older adults in Mexico, the mean number of years of schooling is of 4.6 [16]. Therefore, validation studies of self-administered assessments among older individuals from diverse racial/ethnic backgrounds often beg consideration of the target population’s education level [17]. This is clearly shown in our study, as participants with higher educational levels needed significantly less assistance and completed the assessment faster than those with lower educational levels. Other studies have shown similar results, for example while patients from high-income nations (Australia, Japan, Western Europe, and the US) took a median of 11 min to complete the EORTC-QLQC30 quality of life questionnaire [18], Mexican women (65% with elementary school education or less) required almost double the time (median 19.5 min) [19]. Strategies to assist low-literacy respondents answer self-administered questionnaires include simplifying the language of written materials, using shorter questionnaires, including illustrations and/or graphics, and limiting “sensitive” items [20,21]. However, even materials designed for fifth-grade reading level might be difficult to understand by many patient populations [21].

The reliability and validity of the measures included in this GA have been established by previous studies [2,3,10,22]. This study confirms that the Spanish-language scales are internally consistent and reliable in terms of test–retest within and across methodologies. The IADL score correlated with the ADL score, demonstrating validity as both assess daily function. In addition, our results demonstrate that the Spanish version of the CARG GA is not only reliable in terms of repeated testing, but also consistent across methods of implementation (touchscreen vs. paper/pencil). Although touchscreen technology may lead to fewer missed answers, most participants still preferred more traditional paper/pencil surveys. This might be related to the limited experience with the utilization of touchscreen technologies among older Hispanic adults (85% of the participants in our study said they had no computer skills or were beginners), with <40% of Americans aged ≥70 reporting owning a tablet as of 2020. Interestingly, most participants randomized to the touchscreen version of the GA reported preferring using a tablet over paper/pencil, showing how older adults who are exposed to technology and receive guidance on how to use it are likely to embrace it [23].

There are limitations to this research. First, our sample consisted mostly of women from Mexico, with limited representation of individuals from the Caribbean, Central, and South America. Considering the differences in the Spanish language across countries, it is possible our results may have been different with another sample. However, as greater than half of Hispanics living in the US have Mexican origins, we believe ours to be a reasonably representative sample of Spanish-speaking Americans in southern California [24]. Second, our GA captured if a patient had <9th grade educational level, but a more detailed breakdown regarding education level and health literacy would have provided more information for future clinical application. In addition, while patients were given instructions on how to use the touchscreen methodology, some patients still needed assistance completing the touchscreen GA. The availability of personnel for instruction and extra assistance needs to be considered when planning to utilize an electronic GA. Despite these limitations, this study also has several strengths. Previous studies of self-administered GA tools in oncology included a largely white, non-Hispanic, and college-educated population that may already be comfortable using the touchscreen methodology and understanding the survey items [8,9,10]. This study shows that using self-administered GA tools in oncology might be feasible among older adults with lower educational levels, and also presents researchers with future opportunities for tailoring GA strategies to fit diverse populations.

## 5. Conclusions

This study demonstrates that the Spanish version of the CARG self-administered GA is reliable and consistent in both its touchscreen and paper/pencil format. While the implementation of the GA is also feasible, older adults with lower educational levels may require assistance to complete it. Our results highlight the need for the development, transcultural adaptation, and tailoring of tools for older populations with differing cultural backgrounds, health literacy, and educational levels. As the field of geriatric oncology moves forward and becomes part of everyday oncology practice, research exploring strategies for mitigating racial, ethnic, and social disparities in access to the GA and to GA-guided care, such as the one we report, should take center stage.

## Figures and Tables

**Table 1:** Description of the various scales included in the Spanish Language Geriatric Assessment.

Domain/Measure		Description
**Functional Status**		
	Instrumental Activities of Daily Living (IADL) (subscale of OARS)	Activities required to maintain independence in the community (meal preparation, shopping, making telephone calls, money management). A higher score indicates less need for assistance. (Score range 0–14; No. of items: 7)
	Activities of Daily Living (ADL) (subscale of MOS Physical Health)	Wide range of physical functions (from bathing/dressing to vigorous activities such as running). A higher score indicates a higher level of physical function. (Score range 0–100; No. of items: 10)
	Karnofsky Performance Status (KPS) (Patient-rated)	Global indicator of patient function determined by patient self-report ranging from normal to severely disabled. A higher score indicates a higher level of physical function. (Score range 0–100; No. of item: 1)
	Number of falls in last 6 months	Number of times patient has fallen in last six months (No. of item: 1)
	MOS Social Activity Limitations Measure	Ability to participate in social activities and degree to which health status limits normal social activities. A higher score indicates a better level of social activity. (Score range 0–100; No. of items: 4)
**Comorbid Medical Conditions**		
	Physical Health Section (subscale of the OARS)	Presence of comorbid illnesses. The score is the sum of the present comorbid conditions. (Score range 0–13; No. of items: 13)
**Psychological State**		
	Mental Health Inventory-17 (MHI-17)	Measures the psychological state of patients regarding how the patient has been feeling in the past two weeks. A higher score indicates better mental health. (Score range 0–100; No. of items: 17)
**Social Support**		
	MOS Social Support: Emotional and tangible subscales	Perceived availability of social support. A higher score indicates better social support. (Score range 0–100; No. of items: 12)
**Nutritional Status**		
	Body Mass Index (BMI)	BMI (kg/m^2^) = Weight/height^2^
**Cognition**		
	Blessed Orientation-Memory-Concentration	Gross measure of cognitive function. A score of 11 or greater indicates potential cognitive impairment. (Score range 0–28; No. of items: 6)
**Medications**		
	Number of medications	Number of medications including prescribed, herbal, and over-the-counter medications. (No. of item: 1)

Abbreviations: MOS, Medical Outcomes Study; OARS, Older American Resources and Services. Bold to signify different categories of the geriatric assessment.

**Table 2:** Demographic and disease characteristics for all participants (*n* = 177).

Variable	Overall (*n* = 177)	Group 1 Touchscreen (*n* = 60)	Group 2 Touchscreen & Paper/Pencil (*n* = 56)	Group 3 Paper/Pencil (*n* = 61)
**Sociodemographic Variables**				
Age (Median, Range)	70 (65–95)	68.5 (65–88)	70 (65–95)	70 (65–87)
**Primary Language (*n* (%))**				
Spanish	158 (89.3%)	53 (88.3%)	50 (89.3%)	55 (90.2%)
English	16 (9.0%)	6 (10.0%)	5 (8.9%)	5 (8.2%)
English and Spanish	3 (1.7%)	1 (1.7%)	1 (1.8%)	1 (1.6%)
**Language Spoken at Home (*n* (%))**				
Spanish	138 (78.0%)	50 (83.3%)	45 (80.4%)	43 (70.5%)
English Only/English and Spanish	39 (22.0%)	10 (16.7%)	11 (19.6%)	18 (29.5%)
**Country of Birth (*n* (%))**				
Mexico	119 (67.2%)	38 (63.3%)	42 (75.0%)	39 (63.9%)
Central America/Caribbean	32 (18.1%)	12 (20.0%)	7 (12.5%)	13 (21.3%)
South America	12 (6.8%)	5 (8.3%)	4 (7.1%)	3 (4.9%)
United States	11 (6.2%)	4 (6.7%)	3 (5.4%)	4 (6.6%)
Missing	3 (1.7%)	1 (1.7%)		2 (3.3%)
**Marital Status (*n* (%))**				*
Married or Domestic Partnership	80 (45.2%)	29 (48.3%)	23 (41.1%)	28 (45.9%)
Widowed	41 (23.2%)	16 (26.7%)	16 (28.6%)	9 (14.8%)
Divorced, Separated	36 (20.3%)	8 (13.3%)	13 (23.2%)	15 (24.6%)
Never Married or Missing	20 (11.3%)	7 (11.7%)	4 (7.1%)	9 (14.8%)
**Educational Level (*n* (%))**	*	*		
8th Grade or Less	97 (54.8%)	32 (53.3%)	32 (57.1%)	33 (54.1%)
9–11th Grade	20 (11.3%)	7 (11.7%)	6 (10.7%)	7 (11.5%)
High School Graduate	19 (10.7%)	10 (16.7%)	6 (10.7%)	3 (4.9%)
Bachelor’s or Advanced Degree/Some College/Technical College	39 (22.0%)	10 (16.7%)	12 (21.4%)	17 (27.9%)
Did Not Answer	2 (1.1%)	1 (1.7%)		1 (1.6%)
**Employment (*n* (%))**				
Retired	92 (52.0%)	34 (56.7%)	27 (48.2%)	31 (50.8%)
Homemaker	44 (24.9%)	14 (23.3%)	15 (26.8%)	15 (24.6%)
Disabled/Unemployed/Other	30 (16.9%)	9 (15.0%)	9 (16.1%)	12 (19.7%)
Employed	11 (6.2%)	3 (5.0%)	5 (8.9%)	3 (4.9%)
**Annual Household Income (*n* (%))**				*
<$25,000	78 (44.1%)	24 (40%)	29 (51.8%)	25 (41.0%)
$25,000–$50,000	68 (38.4%)	21 (35%)	19 (33.9%)	28 (45.9%)
>$50,000	23 (13%)	13 (21.7%)	6 (10.7%)	4 (6.6%)
Missing	8 (4.5%)	2 (3.3%)	2 (3.6%)	4 (6.6%)
				
**Clinical Characteristics**				
**Prior Treatment (*n* (%))**				
Surgery	147 (83.1%)	49 (81.7%)	46 (82.1%)	52 (85.3%)
Radiation	99 (55.9%)	33 (55.0%)	30 (53.6%)	36 (59.0%)
Chemotherapy	103 (58.2%)	36 (60.0%)	36 (64.3%)	31 (50.8%)
Hormone Therapy	126 (71.2%)	41 (68.3%)	42 (75.0%)	43 (70.5%)
Targeted Therapy	11 (6.2%)	6 (10.0%)	2 (3.6%)	3 (4.9%)
None	9 (5.1%)	3 (5.0%)	4 (7.1%)	2 (3.3%)
**Number of Prior Lines of Chemotherapy (*n* (%))**				
0	76 (42.9%)	24 (40.0%)	20 (35.7%)	32 (52.4%)
1	78 (44.1%)	28 (46.7%)	28 (50.0%)	22 (36.1%)
2+	23 (13.0%)	8 (13.3%)	8 (14.3%)	7 (11.5%)
**Stage (*n* (%))**				
0-III	146 (82.5%)	46 (76.7%)	49 (87.5%)	51 (83.6%)
IV	31 (17.5%)	14 (23.3%)	7 (12.5%)	10 (16.4%)

* Percentages do not add up to 100 due to rounding. Bold to signify different categories.

**Table 3:** Feasibility of the Spanish-Language Geriatric Assessment for the overall patient population and by method of administration (touchscreen and paper/pencil).

	Overall (*n* = 177)	Touchscreen (*n* = 87)	Paper/Pencil (*n* = 90)	*p*-Value
***n* (%) completed without assistance**	104 (58.8%)	49 (56.3%)	55 (61.1%)	0.52
***n* (%) patients missed any questions**	28 (15.8%)	1 (1.1%)	27 (30.0%)	<0.0001 *
**Time to Finish the GA**				
Median (Range)	28 (8–90)	28 (8–78)	28 (12–90)	0.74 **
Mean (SD)	31.6 (14.0)	32.1 (14.3)	31.2 (13.8)	
**Easiness to Complete GA**				
% Easy/Very Easy	136 (76.8%)	63 (72.4%)	73 (81.1%)	0.17
**Perception of Time Needed to Complete the GA**				
Too Long	29 (16.4%)	15 (17.2%)	14 (15.6%)	0.96
Just Right	135 (76.3%)	66 (75.9%)	69 (76.7%)	
Too Short	10 (5.6%)	5 (5.8%)	5 (5.6%)	
No Answer	3 (1.7%)	1 (1.2%)	2 (2.2%)	
**Computer Skill**				
None	111 (62.7%)	51 (58.6%)	60 (66.7%)	0.19 *
Beginner	40 (22.6%)	25 (28.7%)	15 (16.7%)	
Intermediate	23 (13.0%)	11 (12.6%)	12 (13.3%)	
Advanced	0	0	0	
No Answer	3 (1.7%)		3 (3.3%)	
**Preferred GA Administration Method**				
Paper/Pencil	93 (52.6%)	37 (42.5%)	56 (62.2%)	0.005
Touchscreen	82 (46.3%)	50 (57.5%)	32 (35.6%)	
No Answer	2 (1.1%)		2 (2.2%)	

Abbreviations: GA: geriatric assessment, SD: standard deviation. * *p*-value were obtained from Fisher’s exact test. ** *p*-value was obtained from Kruskal–Wallis test. All other *p*-values were obtained from chi-square test and “No Answer” category was not included. Bold to signify different categories.

**Table 4:** Internal consistency and test–retest reliability of selected measures included in the Self-Administered Spanish-Language Geriatric Assessment.

Scale	Raw Cronbach Alpha	Standardized Cronbach Alpha	Test-Retest Reliability Spearman Rank Correlation Coefficient			
	**All Completed Surveys** **(*n* = 300)**	**All Completed Surveys** **(*n* = 300)**	**Overall** **(*n* = 150)**	**Touchscreen** **(*n* = 50)**	**Paper/Pencil & Touchscreen** **(*n* = 50)**	**Paper/Pencil** **(*n* = 50)**
IADL	0.88	0.88	0.93	0.93	0.95	0.91
ADL	0.93	0.93	0.94	0.93	0.96	0.92
KPS *			0.82	0.81	0.89	0.75
MHI	0.93	0.93	0.88	0.89	0.81	0.88
Social Activity ^†^	0.68	0.69	0.73	0.72	0.72	0.76
Social Support ^‡^	0.96	0.96	0.85	0.82	0.80	0.93

Abbreviations: IADL: Instrumental Activities of Daily Living; ADL: Activities of Daily Living; KPS: Karnofsky Performance Status; MHI: Mental Health Inventory-17. * KPS only has one question so Cronbach Alpha does not apply. ^†^ Medical Outcomes Study Social Activity Limitations Measure. ^‡^ Medical Outcomes Study Social Support Survey: Emotional and Tangible subscales.

**Table 5:** Geriatric assessment scale validity.

	Overall (*n* = 150)	Touchscreen (*n* = 50)	Touchscreen & Paper/Pencil (*n* = 50)	Paper/Pencil (*n* = 50)
	**Spearman Rank Correlation Coefficient**			
IADL with ADL	0.75	0.73	0.75	0.72
IADL with KPS	0.63	0.65	0.69	0.51
IADL with Social Activity	0.47	0.54	0.47	0.41
ADL with KPS	0.71	0.63	0.82	0.66
ADL with Social Activity	0.55	0.63	0.52	0.55
KPS with Social Activity	0.51	0.59	0.46	0.45

Abbreviations: IADL: Instrumental Activities of Daily Living; ADL: Activities of Daily Living; KPS: Karnofsky Performance Status (patient-reported).

## Data Availability

The data presented in this study are available on request from the corresponding author.
